# Modulation of action potentials using PEDOT:PSS conducting polymer microwires

**DOI:** 10.1038/s41598-017-11032-3

**Published:** 2017-09-04

**Authors:** Scott B. Thourson, Christine K. Payne

**Affiliations:** 1 Interdisciplinary Program in Bioengineering, Georgia Institute of Technology, Atlanta, GA 30332 USA; 2 George W. Woodruff School of Mechanical Engineering, Georgia Institute of Technology, Atlanta, GA 30332 USA; 3 School of Chemistry and Biochemistry, Georgia Institute of Technology, Atlanta, GA 30332 USA; 4 Petit Institute for Bioengineering and Biosciences, Georgia Institute of Technology, Atlanta, GA 30332 USA

## Abstract

We describe the use of PEDOT:PSS conducting polymer microwires to modulate action potentials in single cells. PEDOT:PSS conducting polymer microwires are electrochemically synthesized with diameters ranging from 860 nm to 4.5 μm and conductivities of ~30 S/cm. The length of the microwires is controlled by the spacing of the electrodes used for the electrochemical polymerization. We demonstrate the use of these microwires to control the action potentials of cardiomyocytes, showing that the cellular contractions match the frequency of the applied voltage. Membrane integrity assays confirm that the voltage delivered by the wires does not damage cells. We expect the conducting polymer microwires will be useful as minimally invasive devices to control the electrical properties of cells with high spatial precision.

## Introduction

Integration of electronic devices with biological systems requires the development of new, less invasive tools that can modulate cellular activity while minimizing disruption of the surrounding tissue. Conventional electrodes made from metals, silicon, and carbon fibers are relatively hard and brittle making them inherently bio-*incompatible* ^1–3^. The recent development of smaller, more flexible, materials, including single crystalline gold nanowires^4^, nanoneedles, nanopillars, and nanotubes^5–8^, and conformable materials^9–12^ has helped to address the need for less invasive tools. Similarly, conducting polymers have been used as coatings, films, or electrode materials to provide a softer interface with cells^9, 13–18^. Combining the benefits of a wire configuration with the softer material properties of conducting polymers, we have developed conducting polymer microwires as a small, flexible, electrically active material for the bioelectric interface that can be used to modulate the action potentials of individual cells.

Poly(3,4-ethylenedioxythiophene):polystyrene sulfonate (PEDOT:PSS) is a well-characterized conducting polymer^19^, with known biocompatibility^13, 20, 21^, that has been used previously to form conductive^22–24^, flexible (~1 GPa)^25^, nano- to micro-diameter wires. PEDOT:PSS conducting polymer microwires are electrochemically synthesized with diameters ranging from 860 nm to 4.5 μm and conductivities of ~30 S/cm. The length of the microwires (nanometers-millimeters) is controlled by the spacing of the electrodes used for the electrochemical polymerization. We demonstrate the use of these microwires to control the action potentials of cardiomyocytes, showing that the cellular contractions match the frequency of the applied voltage. Membrane integrity assays confirm that the voltage delivered by the wires does not damage cells. Overall, the use of conducting polymer microwires to modulate the action potentials of cardiomyocytes is the first step in the development of a new tool for bioelectric control *in vivo*.

## Results and Discussion

### PEDOT:PSS conducting polymer microwires

**Figure 1:**
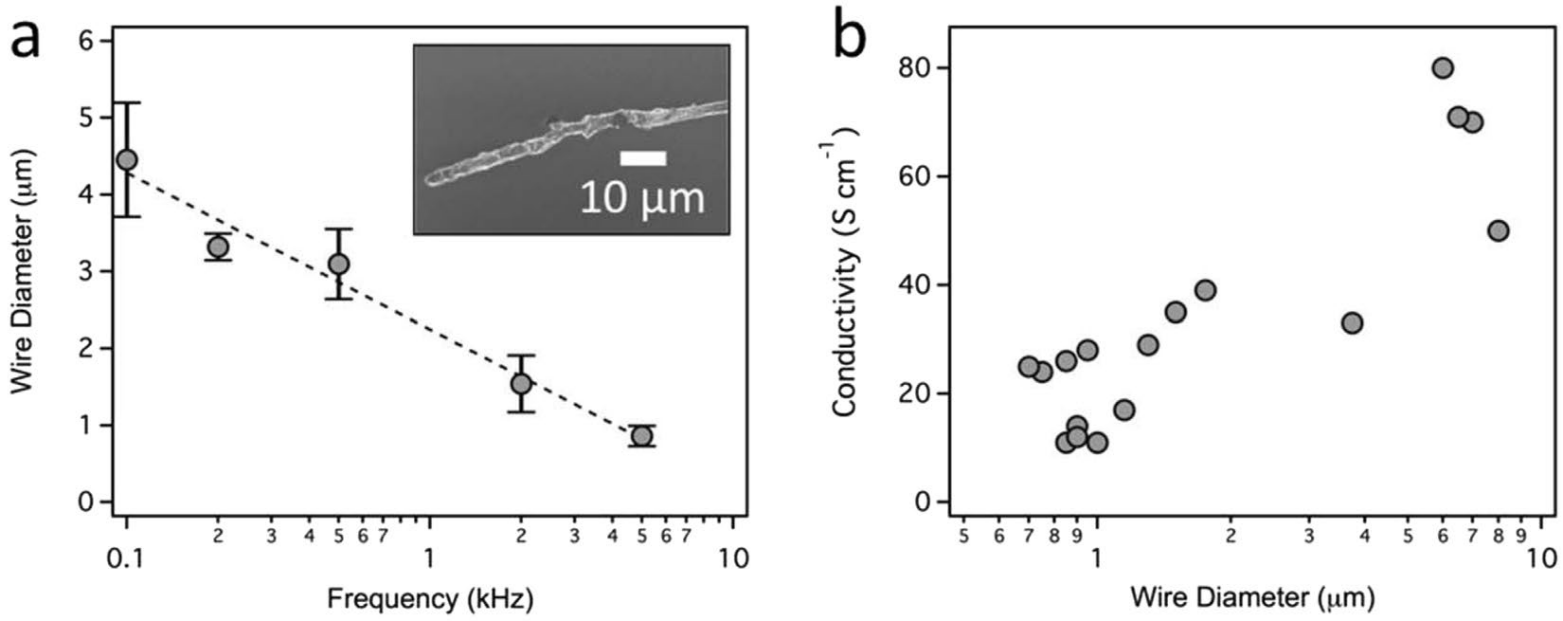
Characterization of PEDOT:PSS microwires. (**a**) Microwire diameter as a function of the AC frequency used for electrochemical polymerization. Data was obtained from SEM images for n ≥ 3 different microwires. The dashed line shows the best fit to the data. Error bars represent ± standard deviation of the mean. The inset shows a representative SEM image of a PEDOT:PSS microwire grown using a 500 Hz square wave. (**b**) Conductivity of PEDOT:PSS microwires as a function of wire diameter. (n = 18, two values overlap).

The electrochemical synthesis of PEDOT:PSS conducting polymer nano- and microwires has been described previously^22–24, 26^. In brief, the wires are grown in an aqueous solution containing EDOT (10 mM) monomer and PSS (20 mM) from the tip of a sharp gold electrode, using a second gold electrode to shape the electric field. The length of the wire is controlled by the spacing of the two gold electrodes during the electrochemical polymerization. Microwire diameter is controlled by the frequency (0.1–5 kHz) of the AC voltage (±1–3 V, square wave) used for the polymerization (Fig. 1a), similar to the approach used for gold and iridium nanowires^27^. Microwire diameters were measured with SEM. Although it is possible to synthesize wires with diameters of 150 nm (100 kHz), micron-diameter wires were used in the experiments described below. Conductivity of the microwires were measured by two-point probe (Fig. 1b) with values ranging from 11 S cm^−1^ to 80 S cm^−1^. Average conductivity for PEDOT:PSS wires synthesized in our lab is 33 ± 21 S/cm (n = 18 wires). Previous work has reported conductivities of ~8.0 S/cm for PEDOT:PSS wires (340 nm diameter) synthesized using an identical approach^23^. The variation in conductivity is likely due to variation in the distribution of the electrically conductive PEDOT and insulating PSS that occurs during the electropolymerization process.

### Modulation of action potentials

**Figure 2:**
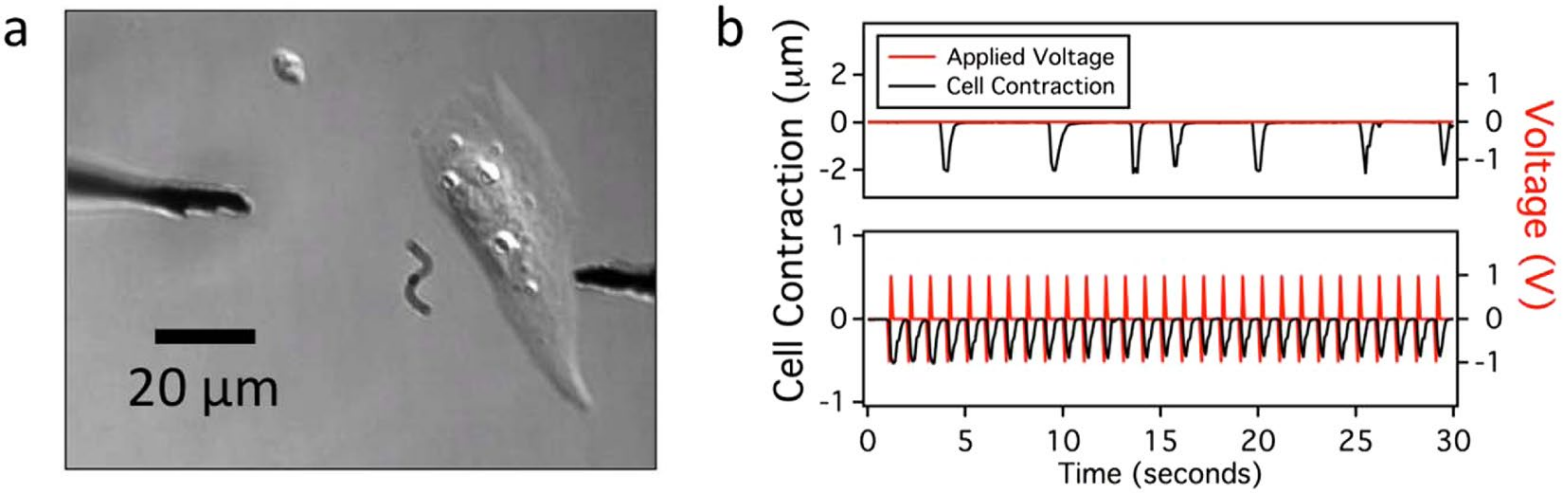
Electrical modulation of a cardiomyocyte. (**a**) Brightfield microscopy image of a neonatal cardiomyocyte showing the two microwires positioned for modulation (3.0 μm diameter, 11 µm long). In this image, the working electrode is on the right. (**b**) Cell contractions are recorded by tracking the displacement of a region of the cell in response to an applied voltage (red, ± 1 V biphasic pulse, 1 ms, 1 Hz). This example is representative of experiments with 40 distinct cardiomyocytes.

The physiological activity of neurons, muscle cells, and heart cells depends on action potentials, rapid changes in ion gradients. For cardiomyocytes, action potentials are associated with cellular contractions, the familiar “beating.” We tested the microwires to determine if they could modulate the action potentials of neonatal rat cardiomyocytes. Induction of an action potential was measured by tracking the displacement of a region of the plasma membrane of individual cells. Two microwires (3.0 μm diameter, 11 μm length), serving as an electrode and counter-electrode, were placed in solution next to a cell of interest (Fig. 2a). In the absence of an applied voltage, there is occasional spontaneous beating, with the cell contracting infrequently (Fig. 2b, top). Applying a ± 1 V biphasic pulse, the cell beats in response to the frequency (1 Hz) of the applied voltage (Fig. 2b, bottom).

**Figure 3:**
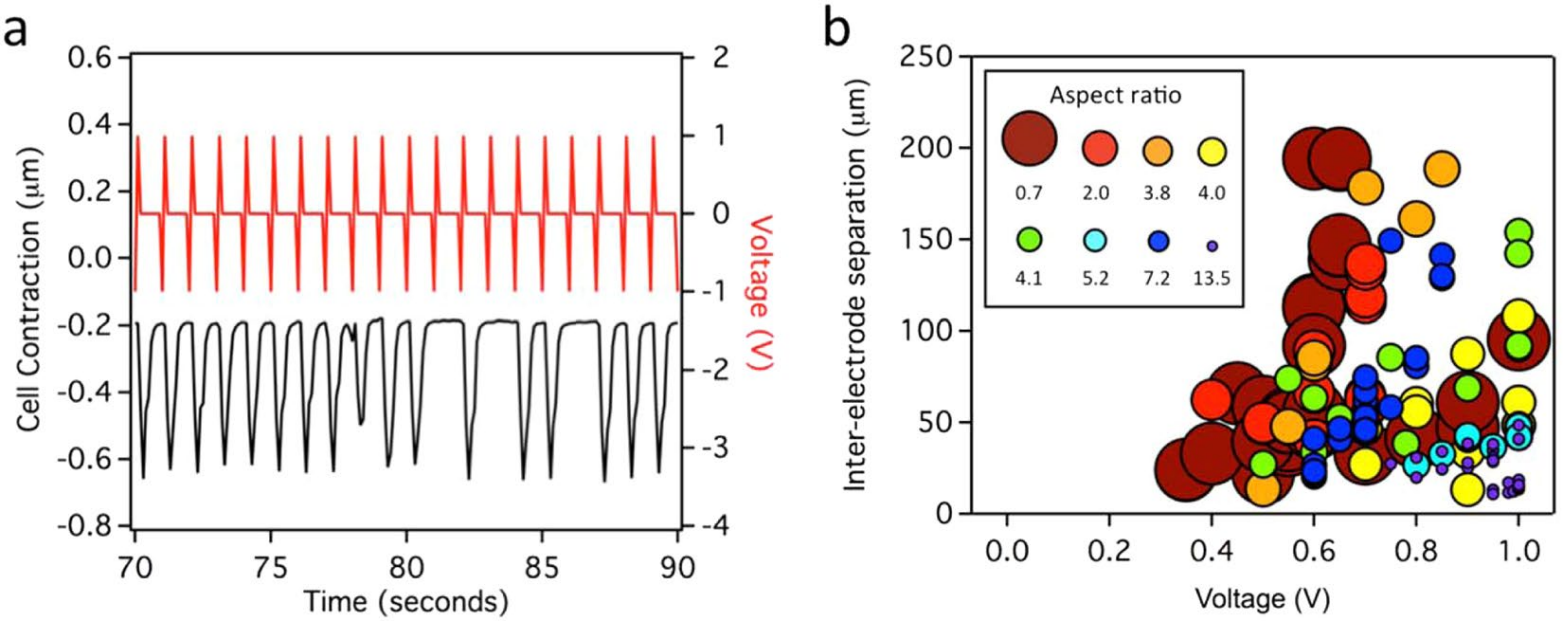
A combination of wire diameter, length, applied voltage, and wire spacing determine whether the electric flux at the cell is sufficient for cellular modulation. (**a**) Successful modulation was determined by observing cell contractions (black) in response to an applied voltage (red), measured using video tracking of a portion of the cell. Insufficient flux resulted in irregular contractions. This example, in which the distance between the wires was increased at 80 s, shows a maximum of 3 consecutive contractions at the increased spacing. (**b**) Modulation data from 8 different wires tested on 19 different cells as a function of aspect ratio (length/diameter) of the working electrode, separation between the wires, and voltage. Circle size represents wire aspect ratio.

Subsequent experiments determined the microwire and electrical parameters necessary to induce an action potential in the cardiomyocytes (Fig. 3). Successful modulation was defined as regular (≥3) contractions in response to the applied voltage, in comparison to skipped, irregular (<3), contractions (Fig. 3a). As expected, modulation is sensitive to microwire diameter, length, spacing, and applied voltage (Fig. 3b). Wire diameter and length can be considered together as an aspect ratio (length/diameter). Spacing is defined as the distance between the two conducting polymer wires serving as electrode and counter-electrode. The distance between the working electrode and the plasma membrane of the cell was held nearly constant (2.31 μm ± 1.32 μm) for all experiments as the modulation of action potentials is very sensitive to this distance. These experiments show that long or narrow microwires require a greater voltage or shorter inter-wire distance to induce an action potential. Ultimately, these three parameters (aspect ratio, spacing, and applied voltage) converge on a minimum electric flux required to induce regular action potentials (Supplementary Fig. 1). Previous studies with cardiomyocytes (chick, guinea pig, and canine, 10 ms stimulus) and conventional bulk electrodes have found that a minimum uniform electric field of 1.4 V/cm – 22.5 V/cm is required for the stimulation of single cells, depending on the cell source and direction of the applied field (parallel or perpendicular)^28–30^. In comparison, an average electric field of 62 ± 19 V/cm was required in our experiments (n = 71 experiments using 19 cells and 8 wires, Figure S1), in good agreement considering the use of a non-uniform electrical field and differences in cell types and experimental approach.

**Figure 4:**
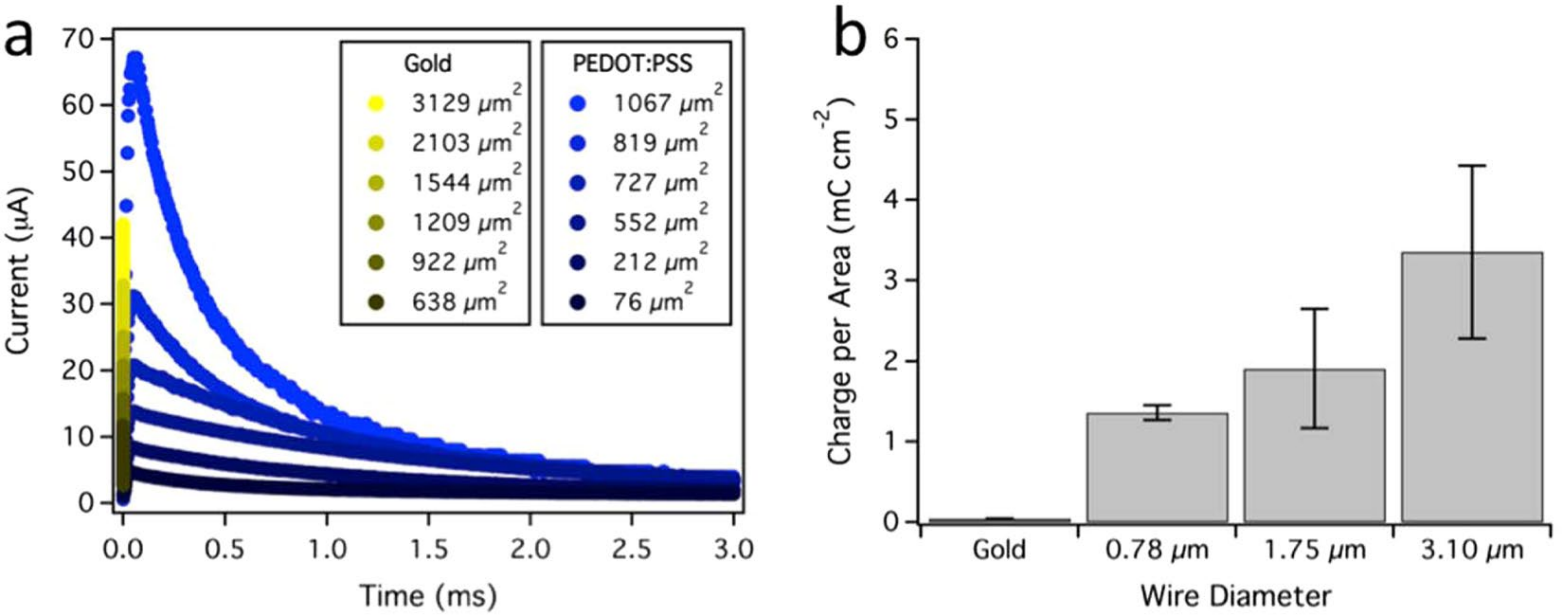
Induction of action potentials requires conducting polymer microwires. (**a**) Representative electrical current transients recorded from a PEDOT:PSS microwire (blue, 3.1 µm diameter) and a gold electrode. Electrical current from a 1 V step was amplified using a transimpedance amplifier and recorded with an oscilloscope. Surface area was varied by controlling the length of microwire or electrode immersed in a buffer solution (Supplementary Fig. S2). (**b**) Charge storage density of a gold electrode and conducting polymer microwires. Charge per area was obtained by integrating the current transients in (**a**) and normalizing them to geometric surface area by approximating the microwire as a cylinder.

We carried out control experiments, testing 13 cells with 7 different pairs of gold electrodes, to ensure that the sharp gold electrodes used to synthesize the microwires were not responsible for the modulation of action potentials. The gold electrodes were not capable of cellular modulation, independent of voltage or spacing between electrodes. To understand this difference, we measured the charge storage density of the conducting polymer microwires and gold electrodes by analyzing current transients in response to a 1 V pulse (Fig. 4). The surface area of each microwire was controlled by immersion into an electrolyte solution of phosphate buffered saline (PBS, Supplementary Fig. S2). Current transients were integrated with respect to time to obtain the total charge transferred by the microwire and normalized by the surface area of the microwire. While the electric field at the sharp tip of the gold electrodes is extremely high (~1 kV mm^−1^ based on COMSOL simulations), when the electric field is multiplied by its small surface area at the cell membrane a very small electric field flux is present, consistent with the inability of these bare gold electrodes to induce action potentials cardiomyocytes.

The measurement of current transients also points towards the minimum electric flux required for cellular modulation. Thinner PEDOT:PSS wires (1.5 μm diameter, 8.5 μm length; 1 V, 1 Hz) did not induce action potentials in the cardiomyocytes. Using a conductivity of 1.5 S m^−1^ to describe the surrounding cell culture media and an electric flux of 2.13 ± 0.65 mV mm (n = 71 experiments using 19 cells and 8 wires, Figure S1), we estimate an instantaneous membrane current of 3.2 μA is required for stimulation, assuming a cellular cross-sectional area of 345 μm^2^.

### Microwire activity does not damage cells

Cell health following the use of microwires was tested using propidium iodide (PI), a fluorogenic dye that binds to nucleic acids. Healthy cells with intact membranes are impermeable to PI, appearing dark in a fluorescence microscopy image. If the cell membrane is damaged, PI enters the cells, leading to a fluorescent cell. Human cervical cancer cells (HeLa) were incubated with PI (500 µM, 10 min pre-incubation) and a voltage was applied to the cells (4.9 µm diameter (average), 4.5–21 μm long wires, ±1 V, 1 Hz, biphasic), similar to conditions used for cardiomyocytes (Fig. 2). We recorded brightfield and fluorescence images before and after 1000 pulses from the microwires. In comparison, experiments with the cardiomyocyte typically measured action potentials for 100 pulses, with each pulse inducing an action potential. No sign of PI internalization was observed, indicating that the microwires and applied voltage do not damage the plasma membrane. We did test the limits of plasma membrane integrity using extremely high voltages (±5 V and ±10 V, Supplementary Fig. S3). At ±10 V (4.9 µm diameter (average), 4.5–21 μm long wires, 1 Hz, biphasic, 60 pulses) some cell death was detected. No cell death was detected at ±5 V following 60 pulses. At longer times and/or higher voltages, electrolysis of water damaged the cells.

## Conclusion

**Figure 5:**
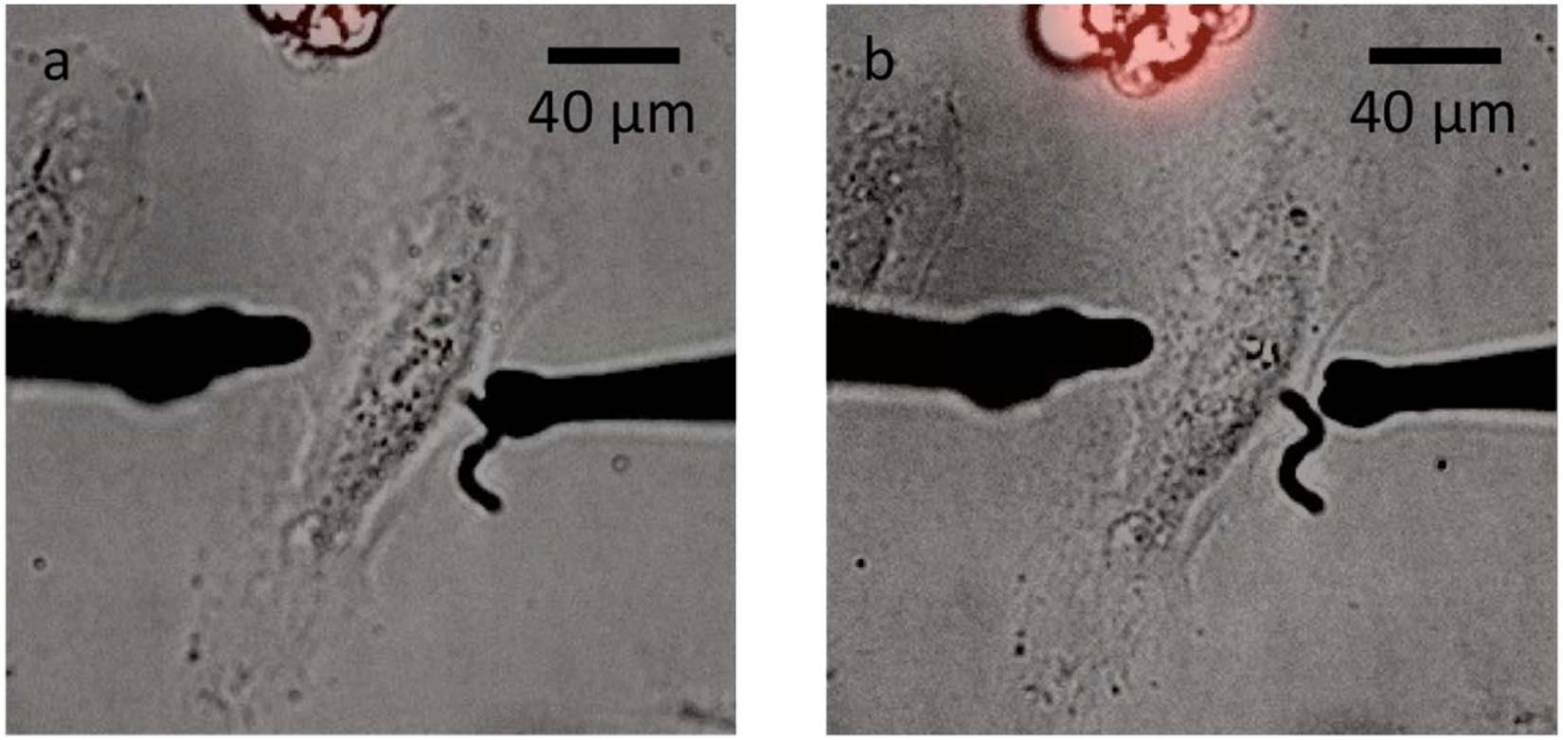
The plasma membrane is not disrupted by microwire activity. (**a**) Overlaid brightfield and fluorescence microscopy images of microwires and a HeLa cell, incubated with PI (500 μM, red), prior to an applied voltage. The cell debris at the top of the image appear red because the plasma membrane is disrupted making them permeable to PI. (**b**) The same cell was imaged after the microwires (left wire = 5 µm diameter, 21 μm long; right wire = 7.5 μm diameter, 4.5 μm long) were used to deliver 1000 consecutive pulses (1 V, 1 Hz, biphasic). Identical experiments were carried out for three different wires, testing 3 cells with each wire (n = 9 cells in total).

These conducting polymer wires, with small, easily tuned diameters (860 nm to 4.5 μm) and moderate conductivities (33 ± 21 S/cm) (Fig. 1) provide a new, much less invasive, tool for the control of action potentials (Fig. 2). In comparison to surface microelectrodes, microwires, positioned by micromanipulators, provide a straight-forward configuration to address single cells. Physically, the conducting polymer wires control the ion concentration at the plasma membrane. They do not penetrate or even contact the plasma membrane. In comparison to patch-clamping, the ability to induce an action potential by only placing the microwires *near* a cell is expected to have a much greater throughput for experiments. The activity of the wires depends on wire diameter, length, spacing, and applied voltage (Fig. 3), which can be considered as a minimum electric flux required for modulation. The electric flux delivered by the microwires does not damage the plasma membrane at the levels required for modulation (Fig. 5). Similar experiments using conducting polymer wires to control the resting membrane potential of *E. coli* also showed that these microwires do not damage cells^26^.

The advantage of the conducting polymer microwires compared to conventional bulk electrodes, which have been used previously to stimulate chick, guinea pig, and canine cardiomyocytes^28–30^, or even patterned substrates, is the small, sub-cellular, diameter of the microwires. Our previous work using similar conducting polymer microwires to control local protein concentration and the resting membrane potential of *E. coli* suggests a <50 μm distance of activity^24, 26^, which will make these microwires useful for cellular-level studies, such as neural mapping, where the localized modulation of an action potential is required. Future work will be necessary to measure the distance of activity both in cell-free systems and for the modulation of action potentials in monolayers of cells. Most similar to the conducting polymer microwires are single crystalline gold nanowires (~100 nm), which have been used for neural recording, including the detection of the site of epileptic spikes,^4^ and the triggered release of dopamine^31^. These gold nanowires have a similar advantage of sub-cellular control and low Young’s modulus, although device construction is difficult. Individual tungsten tips are attached to individual gold nanowires with a conductive carbon paste^4^. In comparison, the conducting polymer wires are synthesized directly from a gold electrode forming the device in a single step. Overall, we expect the conducting polymer wires will provide a new tool for the modulation and mapping of action potentials. The small diameter will allow cellular-level control and the relatively low Young’s modulus (~1 GPa)^25^ suggests they will be a less invasive tool for future *in vivo* studies.

## Materials and Methods

### Electrochemical polymerization and characterization of PEDOT:PSS microwires

The electrochemical synthesis and characterization of PEDOT:PSS nano- and microwires has been described previously^22–24^. In brief, PEDOT:PSS microwires were synthesized from the tip of a sharp gold electrode in an aqueous solution containing 10 mM 3,4-ethylenedioxythiophene monomer (EDOT, Sigma-Aldrich, 483028, St. Louis, MO) and 20 mM polystyrene sulfonate (PSS, Sigma-Aldrich, 243051), used as a counterion. A second gold electrode is used to shape the electric field during the electrochemical synthesis. Fabrication of the sharp gold electrodes from solid gold wire (0.2 mm diameter, 99.9%, Alfa Aesar, 10195-G1) was based on methods used to etch scanning tunneling microscope electrodes^32^, and has been described previously for the electrochemical synthesis of PEDOT:PSS conducting polymer wires^24^. Gold wire was submersed ~1 mm in hydrochloric acid (6 M). Coiled platinum wire (0.3 mm diameter, 99.9%, Alfa Aesar, 43014-BU) served as the counter-electrode. A function generator (Agilent 33120 A) provided a 10 Hz full square wave, ±5 V amplitude. Etching for ~90 s yielded tip diameters <100 nm. After etching, gold electrodes were rinsed with ethanol, then water, and dried under nitrogen. Gold electrodes were plasma cleaned (Harrick) for 15 seconds before use. During microwire synthesis, the gold electrodes were spaced 50 µm apart (tip-to-tip). Polymerization was carried out using a function generator (Agilent, 33120 A, Santa Clara, CA) supplying an alternating, square-wave voltage (0.1 kHz–5 kHz) across the two gold electrodes. Conducting polymer wire diameter was measured using a scanning electron microscope (SEM, Hitachi, SU8230, Tokyo, Japan). Conducting polymer wire length was measured using brightfield microscopy (Olympus IX71, 60x objective, Tokyo, Japan and Andor iXon CCD camera, Belfast, UK). Conductivity was measured with two-point probe using a sourcemeter (Keithley, 2400, Solon, OH) to sweep the voltage between −1 and +1 V while measuring current. Resistance was calculated from the inverse slope of the current-voltage curves. Conductivity was determined using diameter and length measurements obtained from microscopy images.

### Cell culture

Neonatal rat cardiomyocytes were a gift from Prof. Hee Cheol Cho at Georgia Tech and Emory University. Glass bottom cell culture dishes (3.5 cm, MatTek, P35G-0.170-14-C, Ashland, MA) were coated with 40 μg/mL-250 μg/mL fibronectin (356008, Corning, NY) in phosphate-buffered saline (PBS, 14190144, Invitrogen, Carlsbad, CA) for at least 1 hour prior to seeding cells. Culture medium consisted of M199 medium (M4530-1L, Sigma-Aldrich) supplemented with 10 mM HEPES, 0.1 mM MEM non-essential amino acids, 3.5 mg/mL glucose, 2 mM L-glutamine, 4 µg/mL vitamin B12, and 100 U/mL streptavidin/penicillin. This medium was supplemented with 10% FBS for the first two days of culture and 2% for the third day. Cells were used within 3 days of culture (37 °C and 5% carbon dioxide). Prior to experiments, cells were rinsed with Tyrode’s solution (137 mM NaCl, 4 mM KCl, 1.8 mM CaCl_2_, 1 mM MgCl_2_, 10 mM HEPES, 10 mM glucose) at 37 °C.

Human cervical carcinoma cells (HeLa, CCL-2, ATCC, Manassas, VA) were cultured in Minimum Essential Medium (MEM, 61100, Invitrogen) supplemented with 10% fetal bovine serum (FBS, 10437028, Invitrogen) at 37 °C and 5% carbon dioxide. On the day before experiments, MEM with phenol red was replaced with phenol red-free MEM (51200-038, Gibco/Thermo Fisher, Waltham, MA) to reduce background fluorescence. Cells were passaged every 3-4 days. Propidium iodide (PI, P1304MP, Invitrogen) was added to the cells for a final concentration of 500 µM 10 minutes prior to experiments.

### Cardiomyocyte modulation and analysis

Following electrochemical polymerization, PEDOT:PSS microwires were removed from the monomer solution and placed in a cell culture dish containing the cardiomyocytes. Micromanipulators were used to position the microwires. Modulation was carried out in Tyrode’s solution at room temperature. Cathodic-led biphasic voltage pulses were delivered at 1 Hz with a pulse width of 1 ms and an interphase period of 1 ms (Keithley 2400 sourcemeter). Throughout this process, the cells were imaged at 10 fps using brightfield microscopy (Olympus IX71, 60x objective, Andor iXon CCD camera). Contraction of cardiomyocytes was analyzed using Tracker version 4.96 (Douglas Brown, Open Source Physics, http://physlets.org/tracker/index.html) video tracking software.

COMSOL Multiphysics modeling software (COMSOL, Palo Alto, CA) was used to calculate the electric flux at the plasma membrane. The conducting polymer microwires expose cells to a nonuniform electric field. The shape of this field depends on the geometry, conductivity, potential, and position of the PEDOT:PSS wires. These parameters were used in COMSOL simulations to model the instantaneous, nonuniform, electric field generated by each experimentally applied pair of voltage and conducting polymer wire separations. The electric field distribution was integrated over the area of the cell membrane to obtain electric flux. The membrane area was approximated by a rectangle with a height of 5 µm and a width equal to the width of the cell. The rectangular cross section was positioned and angled with respect to the position and axis of the conducting polymer wire to match experimental conditions. The microwire geometry was approximated as a cylinder with a hemisphere located at the microwire tip. The gold electrode was modeled as a cone with the tip placed 5 µm within the base of conducting polymer wire. The length of the conducting polymer wire was defined as the distance from the tip of the gold electrode to the tip of the conducting polymer wire. Electrical conductivity of gold, PEDOT:PSS, and the PBS solution was 456,000 S/cm (COMSOL-provided value), 37 S/cm (approximate average for microwires ≥1 μm diameter), and 1.5 S/m (approximated using a supplier-provided value for PBS), respectively. Voltage boundary conditions were applied at the base of the gold electrode cones. Electric flux values (Supplementary Fig. S1) were obtained for cross-sectional rectangles positioned between 0.25 µm and 48 µm from the tip of the conducting polymer wire near the cell. Rectangular cross-sections were spaced by 1 µm and 2 µm for positions between 0-8 µm and 8-48 µm, respectively.

### Measurement of charge storage density

Electrical current curves of PEDOT:PSS microwires and gold electrodes were measured for 1 V voltage steps. Surface area was controlled using a micromanipulator to position the microwire or gold electrode in a droplet of PBS in electrical contact with a large carbon counter electrode (Supplementary Fig. S2a). Controlling the microwire immersion depth removed the charge contribution from the uninsulated gold electrode allowing us and to measure charge storage as a function of microwire geometry. To prevent the microwires from breaking at the air-water interface, coverslips with a hydrophobic layer of trichloro(1 *H*,1 *H*,2 *H*,2*H*-perfluorooctyl)silane (448931, Sigma-Aldrich) were used to increase the PBS contact angle, allowing the microwire to be inserted and removed from the droplet perpendicular to the air-water interface. A 1 V voltage step was applied to the microwires for 20–30 ms at a frequency of 1 Hz. The current was measured using a current-to-voltage converter constructed using a high-impedance operational amplifier with a gain of 10^5^. The proportional voltage was recorded using an oscilloscope (TBS1064, Tektronix, Beaverton, OR) at a sample rate of 250 kHz. Microscope images were used to measure the microwire immersion depth for each corresponding current transient (Supplementary Fig. S2b). Custom Igor Pro code was used to calculate charge storage by integrating the area under each current transient.

## Electronic supplementary material

Supplementary Information
