# Supplementary material for: Modulation of action potentials using PEDOT:PSS conducting polymer microwires
Source: Sci Rep. 2017 Sep 4;7:10402. doi: 10.1038/s41598-017-11032-3 (PMC5583308; doi:10.1038/s41598-017-11032-3)
Supplement: Supplementary file 1 — Supplementary Information [file 41598_2017_11032_MOESM1_ESM.pdf]

Supplementary Information for:

Modulation of action potentials using PEDOT:PSS conducting polymer microwires

Scott B. Thourson[1,2] and Christine K. Payne[3,4,*]

1. Interdisciplinary Program in Bioengineering, Georgia Institute of Technology, Atlanta, GA 30332, USA. 2. George W. Woodruff School of Mechanical Engineering, Georgia Institute of Technology, Atlanta, GA 30332, USA. 3. School of Chemistry and Biochemistry, Georgia Institute of Technology, Atlanta, GA 30332, USA. 4. Petit Institute for Bioengineering and Biosciences, Georgia Institute of Technology, Atlanta, GA 30332, USA.

*E-mail: christine.payne@chemistry.gatech.edu

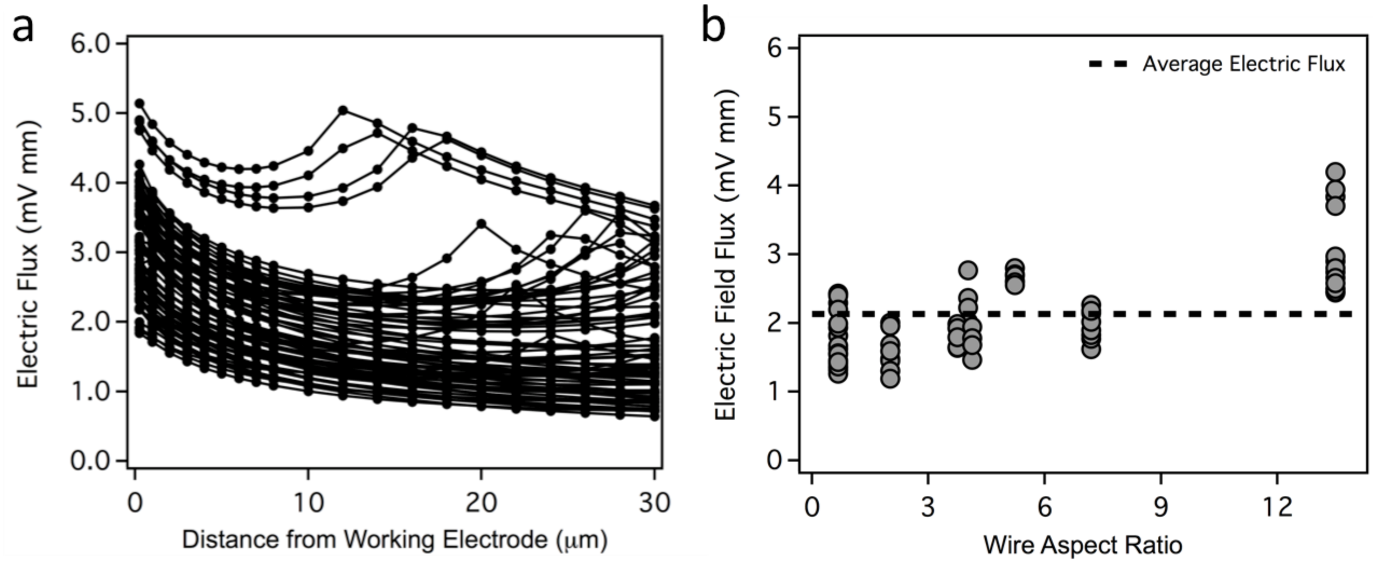

Supplementary Figure S1. The minimum electric flux required for cellular modulation was found by varying the distance between the microwires and determining the point at which cardiomyocyte contractions became irregular (<9 consecutive contractions). 8 different microwires and 19 different cardiomyocyte cells were tested in 71 experiments. The cathode position was held constant. (a) The calculated electric flux (COMSOL) is plotted for each data point in Figure 3b as a function of distance from the working electrode where 0 is at the tip of the cathode. (b) The threshold electric field flux for each curve at 6 μm from the tip of the working electrode is nearly constant (2.13 ± 0.65 mV mm).

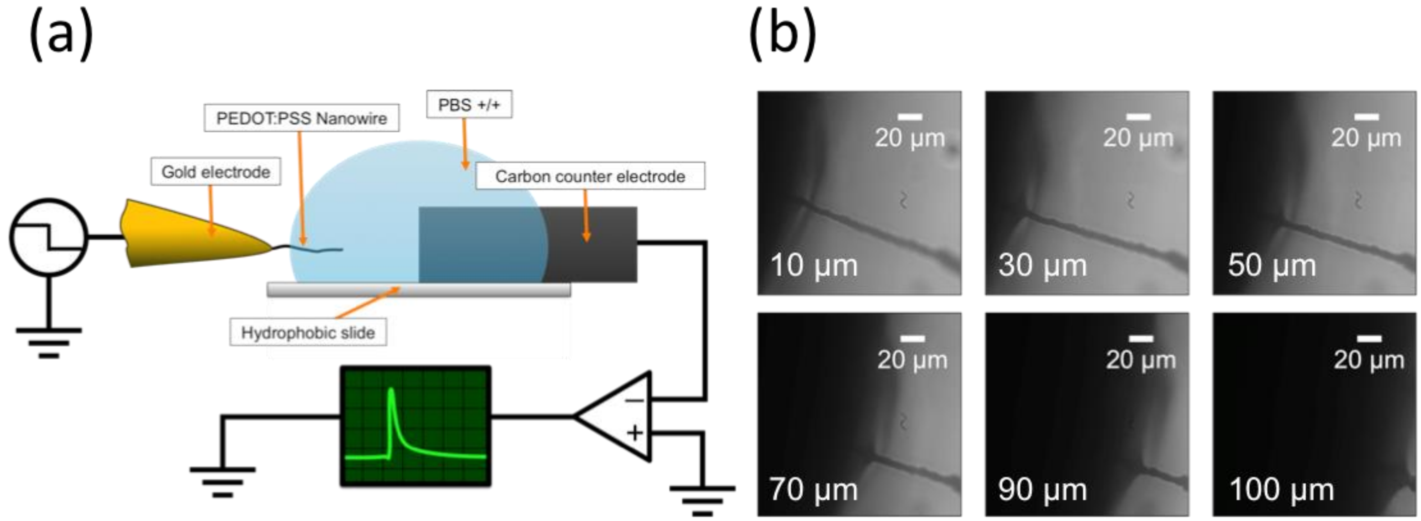

Supplementary Figure S2. Measurement of current transients (Fig. 4). (a) Schematic of the immersion method used to obtain current transients of PEDOT:PSS microwires. Following microwire synthesis, a micromanipulator on an inverted microscope was used to control the electrochemical area of the microwire in a 200 µL drop of phosphate buffered saline (PBS) on a hydrophobic slide. A source meter applied a -1 V cathodic step between the microwire and a large carbon-counter electrode. Current was amplified with a transimpedance amplifier and read using an oscilloscope. (b) Brightfield images showing a 2 kHz microwire immersed at different depths in a PBS droplet.

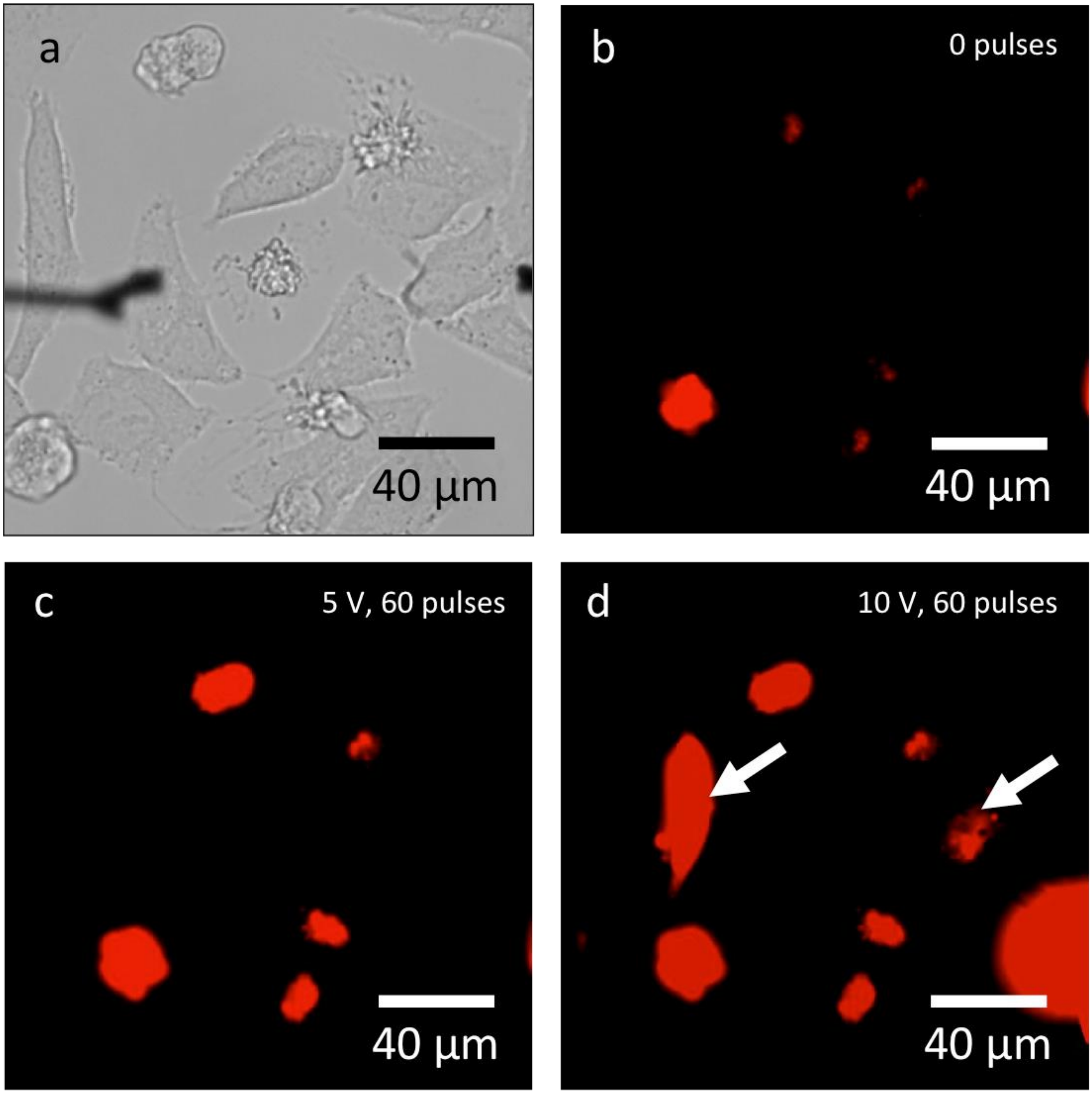

Supplementary Figure S3. Cytotoxicity test of PEDOT:PSS microwire modulation using HeLa cells incubated with propidium iodide (PI). PI is a cell *impermeant* fluorogenic dye that only enters cells or cell debris with a permeabilized membrane, indicating that the cells are damaged. (a) Brightfield microscopy image of PEDOT:PSS microwires placed near the membrane of a HeLa cell and (b) corresponding fluorescence microscopy image with PI (500 μM, red) present in the cell culture medium. The fluorescent signal present in these images shows that the plasma membrane of the cell debris is permeabilized prior to microwire activity. (c) Following 60 pulses at 5 V (1 Hz, biphasic) the cell debris internalize additional PI. The adherent cells remain healthy. (d) After an additional 60 pulses, now at 10 V (1 Hz, biphasic), two dead cells are observed as the new fluorescent signal (indicated by arrows).
